# Role of Polyphenols in Dermatological Diseases: Exploring Pharmacotherapeutic Mechanisms and Clinical Implications

**DOI:** 10.3390/ph18020247

**Published:** 2025-02-12

**Authors:** Juan Salazar, Ángel Ortega, José Luis Pérez, Bermary Garrido, Raquel Santeliz, Néstor Galbán, Maria Paula Díaz, Raquel Cano, Gabriel Cano, Julio Cesar Contreras-Velasquez, Maricarmen Chacín

**Affiliations:** 1 Endocrine and Metabolic Diseases Research Center, School of Medicine, University of Zulia, Maracaibo 4004, Venezuela; juanjsv18@hotmail.com (J.S.); angelort94@hotmail.com (Á.O.); joseluispv2811@gmail.com (J.L.P.); bermarygarrido@gmail.com (B.G.); gabrielasanteliz29@gmail.com (R.S.); nestorag17@gmail.com (N.G.); mariadiazalbornoz@hotmail.com (M.P.D.); 2 Clínica General del Norte, Grupo de Estudio e Investigación en Salud, Barranquilla 080002, Colombia; raquelamiracano@gmail.com; 3 Institut für Pharmazie Königin-Luise, Freie Universität Berlin, Strasse 2-4, 14195 Berlin, Germany; gabriel.simon.cano@fu-berlin.de; 4 Departamento de Productividad e Innovación, Universidad de la Costa, Barranquilla 080002, Colombia; jcontrer30@cuc.edu.co; 5 Centro de Investigaciones en Ciencias de la Vida (CICV), Facultad de Ciencias de la Salud, Universidad Simón Bolívar, Barranquilla 080002, Colombia

**Keywords:** skin disorders, polyphenols, phytotherapy

## Abstract

Although not frequently lethal, dermatological diseases represent a common cause of consultation worldwide. Due to the natural and non-invasive approach of phytotherapy, research for novel alternatives, such as polyphenols, to treat skin disorders is a subject of interest in modern medicine. Polyphenols, in particular, have been considered because of their anti-inflammatory, antitumoral, antimicrobial, and antioxidant properties, low molecular weight, and lipophilic nature that enables the passage of these compounds through the skin barrier. This review discusses the treatment of common dermatological diseases such as acne vulgaris, fungal infections, dermatitis, alopecia, and skin cancer, using polyphenols as therapeutic and prophylactic options. The specific molecules considered for each disorder, mechanisms of action, current clinical trials, and proposed applications are also reviewed.

## 1. Introduction

For decades, dermatologic diseases have occupied relevant positions as main complaints for medical consultation, cataloged by the Global Burden of Disease (GBD) study as the fourth main cause of non-lethal disease and the eighteenth main cause of disability worldwide [1]. These relevant facts transform these entities into important health issues, affecting all age groups and geographical areas [2].

Due to the prevalence and the socioeconomic burden generated by dermatologic diseases, it is imperative to find new therapeutic alternatives to diminish the consequences of these disorders [3]. For the past years, the study of natural polyphenols and their application in dermatologic diseases has obtained a primary interest. This group of molecules is widely distributed in the environment as secondary metabolites from plant sources, characterized by one or more phenolic rings within its chemical structure. Moreover, these molecules have demonstrated notable antioxidant, anti-inflammatory, antimicrobial, and in vivo as well as in vitro anticarcinogenic activities [4,5].

Likewise, due to their low molecular weight and lipophilic properties similar to the stratum corneum, the cosmetic applications of these compounds have been explored, and topical extracts have been prepared, in order to obtain benefits from the direct effect of phenolic compounds when they penetrate the skin´s natural barrier; therefore, these compounds could represent a potential strategy for the prevention and treatment of frequent skin disorders [6,7]. Thus, this review summarizes current preclinical and clinical evidence of polyphenol-based phytochemicals as a treatment for skin disorders (SDs) and the potential molecular mechanisms involved.

## 2. Pharmacotherapeutic Mechanisms of Polyphenols in Main Skin Diseases

### 2.1. Acne Vulgaris (AV)

AV is considered one of the most common chronic dermatologic disorders in the teenage population. This pathology is characterized by its multifactorial etiology, mainly triggered by the proliferation of resident skin bacteria, such as *Propionibacterium acne* and excessive sebum production induced by androgens [8]. In addition, other events, such as the activation of inflammatory cascades leading to hyperkeratinization, increased reactive oxygen species (ROS), and reduced antioxidant activity, provoke hair follicle obstruction, resulting in comedo formation [9]. It is worth mentioning that the high costs and antibiotic resistance observed with conventional treatments have led to the study of numerous plant supplements, which have polyphenols, as an effective therapeutic alternative [10].

In this sense, an anti-inflammatory mechanism of the wild bitter gourd (*Momordica charantia*) total phenolic content leaf extract (TPCLE) has been studied due to its different polyphenolic compounds, such as phenolic acids, myricetin, quercetin, and luteolin as well as its acne-related properties. Clinical evidence indicates that the inhibition of growth of pathogens such as *Propionibacterium acne* is a potential therapeutic target to consider. In consequence, Huang et al. [11] tried to demonstrate this fact through intradermal injections of *Propionibacterium acne*, followed by TPCLE both in vivo and in vitro. TPCLE positively managed to attenuate the secretion of IL-8, IL-1β, and TNFα after inhibiting the expression of the Mitogen-Activated Protein Kinase (MAPK) and, subsequently, the Nuclear Factor Kappa-Light-Chain-Enhancer of activated B cells (NF-kB). Moreover, TPCLE inhibited Matrix Metalloproteinase-9 (MPP-9), an enzyme involved in extracellular matrix degradation and thus in acne lesions, demonstrating the great anti-inflammatory properties of this group of polyphenols [11].

A similar mechanism was found in oregano extracts, *Origanum vulgare* [12], widely distributed in rosmarinic acid, quercetin, carvacrol, and apigenin. In the said mechanism, the inactivation of transcriptional marker signaling responsible for the inhibition of NF-kB was attributed to the inactivation of Toll-Like receptor 2 (TLR2) [12]. Furthermore, the mRNA expression of NOS, ICAM-1, NF-kB, COX-2, and COX-1 [13] was inhibited in human keratinocytes NCTC 2544 with an *M. styphelioides* leaf extract treatment, rich in quercetin, gallic acid, and ellagic acid [14].

Additionally, a flavonoid widely distributed in apples and strawberries, known as phloretin, has been studied for its anti-inflammatory properties and potential antibacterial mechanism. Similarly, phloretin, found in its free form or glycosylated, inhibited the heterodimerization of TLR2/1; however, it also diminished the phosphorylation of the c-Jun N-terminal kinase (JNK) pathway in *HaCaT* keratinocytes stimulated by *P. acnes* in a dose-dependent manner. On the other hand, it was shown that the active site of the 3-ketoacyl-ACP synthase (KAS-III) interacted with rings A and B of the phloretin structure, blocking the reaction between *P. acnes*, KAS-III, and its substrates, and in consequence inhibiting fatty acid synthesis necessary for bacterial survival [10], therefore, proving the anti-inflammatory effect and great antibacterial potential of certain polyphenols.

In contrast, other studies on *Quercus mongolica* leaf extract (QML) examined the antioxidant activity of flavonoids, tannins, triterpenoids, and phenols, mainly pedunculagin (PD), and a decrease in IL-6 and IL-8 production as well as reduced concentrations of Nitric Acid (NA) were observed in the said studies. These phenomena are caused by diminished TLR-4 mRNA. This effect was also demonstrated in a study performed by Kim et al. [15], in which polyphenols of punicalagin, ellagic acid, and pomegranate peels (PPs) were used [16].

Finally, an enzyme regulatory mechanism involved in androgen metabolism has been studied for its role in the pathogenesis of acne. The 5α-reductase type 1 (5αAR-1) is an enzyme directly implicated in androgen-dependent sebum production and a presumptive therapeutic target. For this reason, Koseki et al. [17] attempted to explain the relationship between polyphenols and 5-AR in both in vivo and in vitro studies, using *Bokusoku* (BK), an extract of *Quercus* cortex, extensively found in gallate polyphenols, such as gallotannin and ellagitannin. In this sense, the inhibition of 5AR-1 and, thus, its implication in sebum production suppression were evidenced. Likewise, BK and one of its strongest phenolic compounds, the *Pentagalloylglucose*, suppressed testosterone-induced lipogenesis, blocking androgen-dependent cellular activity. Therefore, the inhibitory activity of 5αAR-1 and lipogenesis suppression are important therapeutic targets that might reduce sebum production in patients with acne [17,18].

### 2.2. Dermatitis

Atopic dermatitis (AD) is a chronic inflammatory disease with a notable allergic component, which can appear from early childhood and is prone to recurrences. This pathology is characterized by clinical manifestations such as erythematous, itchy, and lichenified skin [19]. Its pathophysiology is initiated with an altered genotype, leading to intense immunity and hypersensitivity dysregulations mediated by the increased levels of Th2 and IgE [20]; furthermore, certain environmental and psychological triggers exacerbate AD development [21]. Likewise, intestinal dysbiosis has been implicated as an underlying mechanism of AD since the microbiota shapes immune responses systematically, thus alluding to the complex and multifactorial pathophysiology that comprises this disease [22].

As a consequence of AD recurrence, the chronic use of conventional anti-inflammatory therapy such as topical corticosteroids (TCSs) or calcineurin inhibitors (CIs) are not the safest options due to their known side effects. Moreover, the high economic burden, significant morbidity, and poor quality of life demand new therapeutic alternatives with minimum side effects [23]. Based on polyphenol influence through various anti-AD mechanisms, different polyphenolic compounds studied in preclinical trials promise to be innovative options for this disease.

Furthermore, the pro-inflammatory environment generated by increased cytokine levels has been considered a potential therapeutic target. In light of this, preclinical evidence has shown that diosmetin [24], quercetin, and galanin [25] drive a fall in pro-inflammatory cytokines such as IL-4, TNFα, and IL-1β expression via ERK, p38, and JNK, as well as NF-kB translocation inhibition. In addition, other flavonoids, like taxifolin, aromadendrin, padmatin, and chamaejasmine, have been linked to IgE reduction [26], as well as secondary metabolites of plant polyphenols such as 3-aryl isocoumarins and diarylheptanoids, and these compounds have shown modulatory activities on inflammatory pathways such as the inhibition of NF-kB activation [21,27]. Thus, their efficacy in inhibiting pleiotropic cytokines of Th2 cells that regulate isotype changes from different immunoglobulins to IgE makes these flavonoids an alternative to treat inflammatory responses associated with AD.

Recently, another mechanism to attenuate the pruritus caused by AD has been studied. Nevertheless, the pathophysiological mechanism of AD pruritus is still unknown. Studies show that Thymic Stromal Lymphopoietin (TSLP) is directly linked to pruritus and severeness of AD. Consequently, Fitoussi et al. [28] studied the efficacy of *Tambourissa trichophylla* leaf extract (TTLE), with a titration of >40% polyphenols, mainly troxerutin. Inhibition of TSLP secretion was observed, and the said effect was associated with the IL-1α pathway, a cytokine able to induce the expression of TSLP and therefore alter the cutaneous barrier [29]. Likewise, treatment with systemic resveratrol was associated with diminished TSLP expression in skin lesions similar to EA. Therefore, phenolic compounds seem to be potential therapeutic tools to alleviate AD symptoms such as pruritus.

On the other hand, AD has a close relationship to the intestinal microbiota. Interestingly, AD patients present imbalances between *Clostridium* spp. and beneficial bacteria such as *Bifidobacterium* spp. [30]. In this context, the acacia polyphenol (AP) has been studied, and the topical application of this molecule produced pruritus reduction in DA lesions after inhibiting skin inflammation, and, in consequence, creating a systemic influence in the gut–skin axis. This fact has been demonstrated due to increased beneficial bacteria, such as *Bifidobacterium* spp. and *Lactobacillus* spp., after AP use in murine models with AD [30]. Moreover, a similar behavior was observed after the utilization of *Schizonepeta tenuifolia* Briquet (STB) and *Alpinia oxyphylla* Miquel (AOM) extracts, which are rich in flavonoids and gallic acid, and the said compounds induced changes in the microbiome and therefore supported the importance of the gut–skin axis theory [31].

It is important to point out that, currently, only a few preclinical trials focus on the effects of phenolic compounds in contact dermatitis (CD). Contact dermatitis is characterized by extreme hypersensitivity to allergens once they reach the skin. Thus, Jegal et al. [32] employed *Wikstroemia indica* extract, rich in flavonoids, lignans, and coumarins, to assess their efficacy on CD. Quercetin was strongly associated with the suppression of lysosomal enzyme β-hexosaminidase and IL-4 mRNA expression, which are important regulators of immune responses during the initial phase of CD. Even though more preclinical studies are needed in this field, the potential use of polyphenols as a new therapeutic tool for treating CD and AD is highly relevant.

### 2.3. Skin Fungal Infections

Dermatomycosis comprises a wide range of diseases that target skin, nails, and hair [33]. These are considered the most frequent mycotic infections worldwide and are caused by different etiological agents such as (i) dermatophytes, including the genre *Trichophyton*, *Microsporum*, and *Epidermophyton*, responsible for dermatophytosis and onychomycosis, as well as (ii) yeasts responsible for cutaneous candidiasis and pityriasis versicolor, such as *Candida* spp. and *Malassezia* spp., respectively [34]. A common feature among fungal pathogens of the skin is the production of keratinolytic enzymes responsible for the degradation of the keratinized epithelium and induction of inflammatory responses in the site of infection [35]. The aforementioned process depends on the intensity of inflammatory mediators produced by the strains involved and the host’s response to the pathogen [36].

**Figure 1:**
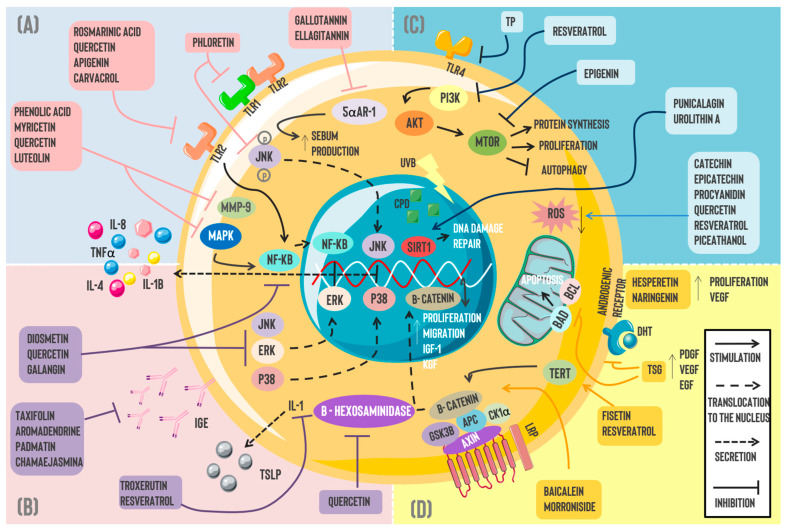
Pharmacotherapeutic mechanisms of polyphenols in skin disorders: Currently, multiple pharmacological mechanisms have been attributed to naturally occurring polyphenols for different skin pathologies, including (**A**) *Acne vulgaris*, where the actions of gallotannin and ellagitannin suppressed sebum production via 5αAR-1 inhibition. Moreover, phloretin’s anti-inflammatory properties inhibit TLR2/1 heterodimerization and JNK signaling in human keratinocytes. Phenolic acid, myricetin, quercetin, and luteolin can inhibit MMP-9 and, thus, diminish acne lesions and, in addition, have similar effects to rosmarinic acid, apigenin, and carvacrol over NF-kB by blocking the molecule and decreasing the subsequent IL-8, II,-1B, and TNFα secretion; (**B**) the anti-inflammatory effect of polyphenols is observed in dermatitis where diosmetin, quercetin, and gallatannin inhibit signaling pathways such as ERK, JNK, and p38, as well as NK-kB translocation to the nucleus. Likewise, flavonoids such as taxifolin, aromadendrin, padmatin, and chamaejasmine were linked to IgE decrements. Resveratrol and troxerutin decrease TSLP expression, and this effect is likely associated with the IL-1α pathway. Quercetin has been associated with *β*-hexosaminidase inhibition. (**C**) Skin cancer is linked to ROS overproduction, DNA damage, and UV ray effects. Certain polyphenols such as catechin, epicatechin, procyanidin, quercetin, resveratrol, and piceatannol have demonstrated antioxidant properties by diminishing ROS levels. On the other hand, resveratrol and epigenin possess antitumoral activity through direct or indirect mTOR blockade. A similar effect is observed by inhibiting TLR4 through TP. Punicalagin and urolithin A repair DNA damage induced by UV rays via SIRT1 stimulation. (**D**) Regarding alopecia, morroniside and baicalein induce cellular proliferation as well as migration through the Wnt/β catenin pathway. Furthermore, fisetin and resveratrol promote β-catenin translocation and higher IGF-1 and KGF expression through a TERT-dependent mechanism. Lastly, polyphenols such as hesperetin and naringenin stimulated VEGF expression and proliferation in DPCs. Simultaneously, this molecule promotes PDGF and EGF increments, inhibiting the DHT effect on androgenic receptors. Similarly, TSG diminishes DPC apoptosis via Bcl-2/BAD blockade. Abbreviations: TLR: Toll-like receptor; JNK: Jun N-terminal kinase; 5αAR-1: 5-alpha reductase; MMP-9: Matrix Metallopeptidase 9; NF-KB: Nuclear Factor Kappa-Light-Chain-Enhancer of activated B cells; MAPK: Mitogen-Activated Protein Kinase; ERK: Extracellular-regulated Kinase; IL: Interleukin; TNFα: Tumor Necrosis Factor Alpha; IgE: Immunoglobulin E; TSLP: Thymic Stromal Lymphopoietin; TERT: telomerase reverse transcriptase; TSG: 2,3,5,4′-tetrahydroxyestilbene-2-*O*-β-d-glucoside; PDGF: Platelet-derived Growth Factor; VEGF: Vascular Endothelial Growth Factor; EGF: Epidermal Growth Factor; DHT: Dihydrotestosterone; BAD: BCL 2-associated agonist of cell death; BCL: B-cell Lymphoma; IGF: insulin-like growth factor; KGF: keratinocyte growth factor; ROS: reactive oxygen species; TP: tea polyphenol; PI3K: Phosphoinositide 3-Kinase; AKT: Protein Kinase B; mTOR: The Mechanistic Target of Rapamycin; UVB: ultraviolet B; CPD: cyclobutane pyrimidine dimer; SIRT: Sirtuin.

In this context, an effective approach to treat dermatomycosis involves adding topical corticosteroids to an antimycotic therapy to diminish initial inflammatory symptoms and avoid bacterial superinfection. However, the risk of side effects and its high cost are difficulties worth considering [37]. Recently, different phenolic compounds have been proposed as an alternative to this scenario since these molecules can interfere directly with the growth and survival of the fungal species, attenuate inflammatory responses, and regulate ROS production (Figure 1) [37].

The effect of polyphenols on fungal growth occurs through various cellular targets. The inhibition of ergosterol biosynthesis has been documented as one of the mechanisms, considering that this blockade constitutes a key factor that leads to alterations in the plasmatic membrane and a consequent loss of cellular integrity [38]. Along with this perspective, it has been demonstrated that gallic acid (GA) [39] and ellagic acid (EA) [39,40] significantly diminish the ergosterol content in the membrane of *T. rubrum* in vitro by reducing the activity of limiting enzymes of sterol synthesis: sterol 14α-demethylase P450 (CYP51) and squalene epoxidase (SE). This potential antifungal activity of both compounds is equivalent to conventional treatments such as fluconazole and terbinafine.

Similarly, caffeic acid (CA) and Licochalcone A (LicoA) exhibit a powerful antimycotic effect on *T. rubrum*, with a minimum inhibitory concentration (MIC) of 86.59 and 11.52 μM, respectively. The mechanism involved encompasses an inhibition of gene expression related to ergosterol biosynthesis, plasmatic membrane synthesis, and glyoxylate cycle, with the latter process significantly increased with LicoA, suggesting a multimodal role of these agents, which act by interfering with key pathways of *T. rubrum* survival [41]. An additional therapeutic goal to consider is the fatty acid synthase (FAS), whose inhibition reduces fatty acid synthesis and, consequently, alters the mycotic membrane lipidic content [42]. In this sense, the antifungal action of flavonoids, such as gallate of epigallocatechin [43], quercetin, and trans-chalcone [44,45] via FAS blockade, has been reported.

In addition to the effect on fatty acid metabolism, the action of trans-chalcone over different virulence factors produced by *T. rubrum* has been described. In line with this, the downregulation of genes that codify proteases and heat shock proteins (Hsp70, Hdp88, and Hsp90) has been observed. Likewise, this flavonoid can interact with MAPK, Tor, and CDK signaling pathways, which mediate the assembly and maintenance of the plasmatic membrane. These findings suggest a role for the aforementioned compound in several aspects of dermatophyte biology that should be further explored in future investigations [46]. In contrast, Gaziano et al. assessed in silico and in vitro anti-dermatophytic effects of phenolic compounds extracted from *Cardiospermum halicacabum.* Evidence indicated that rutin and luteolin directly affect the growth of *T. rubrum*, in a potential mechanism mediated through the interaction with the ATP domain of *Hsp90*. Interestingly, although both compounds showed a particular effect, it was not superior to the effect demonstrated by the total leaf extract. Thus, it can be inferred that using *Cardiospermum halicacabum* extract could represent a new antifungal strategy to explore as a single agent or in combination with conventional drugs [47].

On the other hand, baicalein and wogonin, two flavones present in root extracts of *Scutellaria baicalensis*, exert a potential antifungal activity against *T. rubrum*, *T. mentagrophytes*, *A. fumigatus*, and *C. albicans*. Moreover, the powerful effect of baicalein against the previously mentioned pathogens has been observed; meanwhile, wogonin inhibited the activity of every pathogen except *C. albicans.* The mechanism of action is associated with increments in mitochondrial ROS and, therefore, the induction of modifications in mitochondrial membrane potential (MMP), which results in cellular apoptosis [48]. Likewise, isoquercitrin (ISO) exerts a synergic effect with fluconazole and amphotericin B, promoting cellular apoptosis and altering the permeability of *C. albicans* membrane through the inhibition of Superoxide Dismutase (SOD) mitochondrial activity, leading to increments in oxidative stress levels [49].

Lastly, the anti-inflammatory potential of some phenolic compounds represents an attractive therapeutic target to explore in dermatomycosis. In this sense, Gomes et al. evaluated the anti-dermatophytic effect of *Salacia senegalensis* leaf extract, observing the antifungal activity of the plant against *T. rubrum* and *E. floccosum*, and these effects could be partially attributed to the presence of flavanols with potential synergic actions, such as quercetin, myricitrin, and quercitrin. Likewise, the anti-inflammatory effect of the extract stands out in a mechanism dependent on eicosanoid derivates of 5-lipooxygenase (5-LOX). Hence, studying flavonoids in *Salacia senegalensis* extract might represent an alternative to consider when it comes to reducing acute inflammatory responses associated with dermatophytes [50].

### 2.4. Alopecia

The social stigma generated from hair loss (HL) has made this condition a common complaint in dermatologic appointments [51]. From a clinical perspective, alopecia possesses different etiologies. Androgenic alopecia (AGA) is the most frequent, affecting men and women through different stages of life, with elevated dihydrotestosterone (DHT) levels as its main contributing factor [52]. Furthermore, AGA is a non-scarring alopecia characterized by rounded, flat, alopecic patches and caused by the autoimmune destruction of the hair follicle (HF) [53]. Finally, telogen effluvium (TE) is a heterogeneous entity characterized by sudden and diffuse hair loss that occurs around three months after a triggering event, as a consequence of regular hair growth cycle alteration, abruptly transitioning from the anagen phase (active growth) to telogen phase (rest) [54].

Despite the growing interest in finding new therapeutic strategies to treat HL in recent years, the available pharmacological options are still scarce. The only drugs currently approved by the FDA to manage this condition are minoxidil and finasteride. In addition, due to limited efficacy and potential side effects, the use of these drugs remains controversial [55]. On the contrary, phenolic extracts have emerged as a safer and more economical alternative to treat HL. Significant effects of epigallocatechin [56], quercetin [57], and baicalein [57], among others, have been documented in diverse experimental models.

Several hypotheses have been developed to clarify the potential mechanisms of action of polyphenols to induce hair growth. An attractive target explored is the regulation of hair follicle dermal papilla cell (DPC) activities, a specialized cellular group that controls the proliferation and regeneration of hair follicles by producing different growth factors [58]. Studies indicate that hesperetin and naringenin perform important roles via the stimulation of human DPC proliferation in vitro as well as cellular protection from oxidative stress mediated by hydrogen peroxide (H_2_O_2_) and increments in VEGF, which is an important angiogenic factor, capable of promoting the restoration of oxygen supply within the growing HF [59].

Furthermore, baicalein exerts a promising role in the modulation of DPC activities. It has been documented that the topical application of this flavonoid promotes follicular growth in Balb/c-null mice through a potential Wnt/β-catenin pathway-dependent mechanism and alkaline phosphatase (ALP) activation in this cellular population [60]. Likewise, this mechanism has been demonstrated with other compounds and cell types. It has been observed that a glycoside extracted from the medicinal plant *Cornus officinalis* known as morroniside can stimulate the proliferation and migration of the outer root sheath cells (ORSCs), as well as induce HF growth in vitro through the Wnt/β-catenin signaling pathway in a dose-dependent manner. Thus, the regulation of this pathway constitutes a common mechanism used by phenolic extracts to induce the onset of anagen phase and follicular growth [61].

On the other hand, the prolongation of DPC viability represents a key approach to avoid the transition to telogen phase and, as a consequence, attenuate HL [62]. Accordingly, Shin et al. demonstrated that *Polygonum multiflorum* (PM) extract, whose main component is 2,3,5,4′-tetrahydroxyestilbene-2-*O*-β-d-glucoside (TSG), induces an anti-apoptotic effect in human cultured DPCs through the regulation of Bcl-2/BAD, thus extending the anagen phase. Moreover, the extract promotes increments in the production of VEGF, PDGF, and EGF and inhibits the effect mediated by DHT on androgenic receptor (AR) expression. This multimodal mechanism of PM extract on hair growth might represent a potential approach to treating AGA; however, new studies to explore this phenomenon with individual intake of TSG are required [63].

Lastly, fisetin and resveratrol have played a key role in the transition from the telogen to anagen phase and, consequently, in dorsal skin hair growth in mice. In a mechanism involving the telomerase reverse transcriptase, whose overexpression induced β-catenin nuclear translocation, increments in cytokines such as insulin growth factor-1 (IGF-1) and keratinocyte growth factor (KGF), as well as the promotion of stem cell proliferation within the hair follicle, were observed. Therefore, this positive effect of TERT on the anagen phase, induced by these polyphenols, represents a therapeutic mechanism to consider in future investigations [64].

### 2.5. Skin Cancer

Skin cancer is one of the most common cancers worldwide. The number of new cases has increased alarmingly over the last decade. This increase could be attributed to continuous exposure to sunlight and the ozone layer changes that predispose the earth’s surface to higher ultraviolet (UV) radiation [65]. Skin cancer is classified into two subtypes: (i) Non-Melanoma Skin Cancer (NMSC), also known as keratinocyte cancer, which represents the most common malignant neoplasia, with Basal Cell Carcinoma (BCC) and squamous cell carcinoma (SCC) comprising 99% of every NMSC [66], and (ii) Melanoma Skin Cancer (MSC), even though it only represents 3% of all the skin cancer cases, it has the highest mortality rate and poorer prognosis [67].

The economic burden of skin cancer on the health system worldwide has also increased [68], boosting the research to find new, more economical and safer therapeutic strategies. In this context, it has been noted that polyphenols have significant effects on cancer progression through the modification of signaling cascades affected during carcinogenesis [69], proliferation, migration, and invasion [70], as well as angiogenesis [69], therefore improving patients’ prognosis.

UV rays can penetrate the epidermis and reach the superior dermis to induce biological side effects, including oxidative stress, inflammation, DNA damage, and premature photoaging [71]. DNA damage results in cyclobutane pyrimidine dimer (CPD) formation. These molecules act as promoters of immunosuppression and trigger photocarcinogenesis [72]. Furthermore, the potential of phenolic compounds extracted from pomegranate (*Punica granatum*), such as punicalagin and urolithin, as DNA repair tools has been documented. These molecules possess powerful properties as SIRT1 inducers, facilitating Nucleotide Excision Repair (NER) through the deacetylation of XPC and XPA genes, finally eliminating previously formed CPDs [73].

Currently, it is known that reactive oxygen species have a major role in cutaneous carcinogenesis via the overactivation of multiple pathways such as MAPK, phosphatidylinositol 3-kinase (PI3K)/kinase protein B (AKT)/mammalian target of rapamycin (mTOR), and NF-kB pathway [74]. Likewise, clinical evidence highlights the bioprotector effect of *Arachis hypogaea* (peanut) polyphenols, which include catechin, epicatechin, procyanidin, and quercetin, against oxidative stress due to their capacity to diminish ROS levels [75]. Moreover, Eskandari et al. monitored reactions between hydrogen peroxide, quercetin, resveratrol, and piceatannol on the skin through an oxygen electrode covered with a cutaneous membrane (SCOE) as a tool in vitro, demonstrating that these polyphenolic compounds promote antioxidant reactions in 2–22 min [76].

In addition, polyphenols, such as resveratrol, have shown antiproliferative effects on tumor cells due to the downregulation of phosphorylated PI3K and AKT [77]. Similarly, epigenin, a natural flavonoid, has proven to inhibit skin cancer induced by UV rays via mTOR blockade, improving keratinocyte autophagy and diminishing its proliferation [78]. These antitumoral effects were observed in an in vitro and in vivo study performed by Chen et al., where tea polyphenols (TPs) showed time- and dose-dependent effects over melanoma cells, significantly inhibiting their proliferative, migrative, and invasive capacity through TLR4 suspension [70].

Polyphenols could contribute to skin cancer prevention and treatment through the induction of malignant cell apoptosis; as highlighted in Arumugam et al.’s preclinical trial, the synergic use of polyphenols such as resveratrol, epigallocatechin-3-gallate, and diallyl trisulfide could play a significant role in apoptosis inhibition. In the said study, A431 cells were used in a human epidermoid carcinoma model, and the application of polyphenolic compounds induced apoptosis inhibition via Bax and Bad upregulation, as well as pre-apoptotic proteins, while molecules such as Bcl2 and caspases 3 and 9 were downregulated [79].

### 2.6. Rosacea

**Figure 2:**
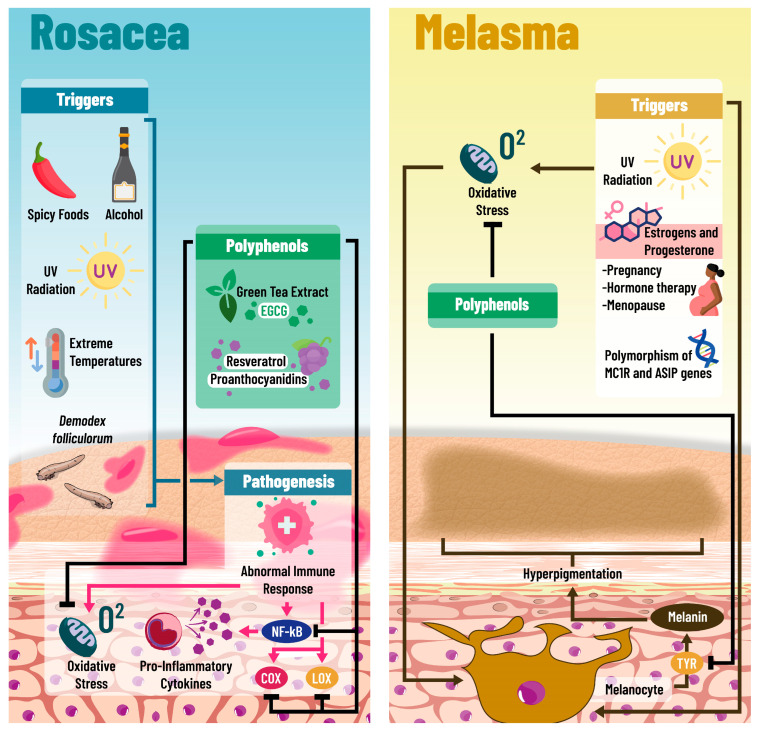
Underlying mechanisms of polyphenols in rosacea and melasma: The pathophysiological basis of rosacea is not completely understood; however, multiple triggers have been identified; among these are the overexposure to the acarus *Demodex folliculorum*, extreme temperatures, UV light exposure, and the consumption of alcohol and spicy foods. All of these elements can promote abnormal responses on the immune system, conditioning a pro-inflammatory state on the skin, characterized by NF-kB expression, pro-inflammatory cytokine secretion, and increased oxidative stress within the cells. It has been observed that polyphenols such as EGCG, resveratrol, and proanthocyanidins may interfere with the inflammatory response of these patients, inhibiting NF-kB expression, pro-inflammatory cytokine secretion, and Cox and Lox activation. Furthermore, because of their antioxidant activities, polyphenols are able to reduce oxidative stress levels. Likewise, polyphenols seem to improve melasma, and this condition is characterized by skin hyperpigmentation and is associated with genes such as MCR1 and ASIP, due to the activation of melanocytes by various factors like UV light and hormones (estrogen and progesterone) in pregnant women and menopausal women receiving hormone therapy. The potential protective mechanisms of polyphenols in melasma are oxidative stress reduction, inflammatory pathways’ downregulation, and potential tyrosinase inhibition. Abbreviations: COX: cyclooxygenase; LOX: lipoxygenase; NF-kB: Nuclear Factor Kappa-B; TYR: Tyrosine; UV: ultraviolet; EGCG: epigallocatechin gallate.

Rosacea is a chronic inflammatory dermatosis affecting the central face, characterized by persistent erythema, telangiectasia, and papulopustular eruptions. Although its etiology is multifactorial, including genetic and environmental influences, the exact pathophysiological mechanisms remain elusive (Figure 2).

Rosacea is a prevalent inflammatory skin condition predominantly affecting the central face, with symptoms that significantly deteriorate the patient’s quality of life [80,81]. It manifests primarily as facial flushing, erythema, telangiectasia, and papules/pustules. The pathogenesis of rosacea is complex and not fully understood, but it likely involves a combination of genetic predisposition and environmental triggers.

Rosacea presents in four distinct subtypes, each with unique clinical features: 1-Erythematotelangiectatic Rosacea (ETR) characterized by persistent facial redness, flushing, and visible blood vessels; 2-Papulopustular Rosacea (PPR) which features small, inflammatory papules and pustules on a backdrop of erythema; 3-Phymatous Rosacea involves skin thickening and irregular surface nodules, most often on the nose (rhinophyma); and 4-Ocular Rosacea that affects the eyes, leading to irritation, redness, and watery eyes [82].

The pathogenesis of rosacea is complex, including an overabundance of *Demodex mites*, particularly *Demodex folliculorum*, associated with triggering inflammatory reactions contributing to rosacea. Evidence suggests that rosacea patients exhibit abnormal immune responses, including increased levels of pro-inflammatory cytokines that contribute to skin inflammation and damage. Environmental triggers such as UV radiation, extreme temperatures, spicy foods, and alcohol have been recognized for exacerbating rosacea symptoms [82].

Emerging research suggests that antioxidants and polyphenols, which can neutralize free radicals and reduce oxidative stress, have potential clinical benefits in rosacea treatment. Polyphenols exert anti-inflammatory effects, potentially attenuating inflammatory pathways involved in rosacea. They also enhance the skin’s barrier function, which helps protect against environmental irritants. Notable polyphenols with proven efficacy in rosacea management include green tea extract, which contains epigallocatechin gallate (EGCG), known for its strong anti-inflammatory and antioxidant effects. Resveratrol, found in grapes and berries, offers significant anti-inflammatory and antioxidant benefits, making it beneficial in reducing rosacea symptoms. Grape seed extract is known for its high proanthocyanidin content, which has anti-inflammatory and antioxidant capabilities [83].

Aside from polyphenols, rosacea management includes topical treatments such as metronidazole, azelaic acid, and ivermectin, which are widely used. Oral antibiotics like doxycycline and tetracycline are prescribed for more severe cases. Laser and light therapies target vascular and inflammatory components of rosacea, offering symptom improvement [84].

Rosacea is a complex dermatological condition that significantly impacts patients’ lives. While the exact cause of rosacea is still under investigation, integrating polyphenols into treatment regimens offers a promising approach due to their antioxidative and anti-inflammatory properties. Comprehensive management, incorporating established and emerging therapies tailored to individual patient needs, remains paramount for effectively controlling and improving rosacea symptoms.

Moreover, the role of polyphenols in dermatology, particularly in the context of inflammatory skin diseases like rosacea, extends beyond their antioxidative and anti-inflammatory properties. Polyphenols have been shown to inhibit the activation of NF-kB, a key transcription factor in the inflammatory response, which leads to the downregulation of various pro-inflammatory cytokines and mediators involved in rosacea [85]. Additionally, polyphenols can modulate the activity of enzymes such as cyclooxygenase and lipoxygenase, further reducing the synthesis of pro-inflammatory eicosanoids, i.e., lipid compounds that exacerbate inflammation. This multifaceted mechanism of action makes polyphenols an attractive component in the holistic management of rosacea, offering potential synergies with other therapeutic modalities to enhance treatment efficacy and patient outcomes [85].

### 2.7. Melasma

Melasma, an acquired facial hyperpigmentation, predominantly affects women of darker skin and is characterized by symmetrical hyperpigmented patches typically found on the forehead, cheeks, upper lip, and chin, significantly impacting the quality of life [85,86]. These patches can cause emotional distress and affect social interactions, making a thorough understanding of the pathogenic mechanisms and therapeutic options crucial for effective management.

The etiology of melasma is considered multifactorial, involving genetic, hormonal, and environmental components. Genetic predisposition plays a significant role, with a strong concordance observed among twins and family members, and polymorphisms in genes related to melanogenesis, such as MC1R and ASIP, have been linked to an increased risk. Hormonally, estrogen and progesterone, which regulate melanogenesis, are crucial, with a higher prevalence of melasma noted during pregnancy, menopause, and among users of oral contraceptives or hormone therapy. Environmentally, UV radiation is a primary trigger, stimulating melanin production and exacerbating hyperpigmentation. Other factors like heat, stress, and certain medications also contribute to the onset or worsening of melasma [87,88].

Several risk factors increase the likelihood of developing melasma, including female gender, darker skin phototypes, a family history of melasma, hormonal fluctuations during pregnancy or due to hormone therapy, and excessive UV radiation exposure [88].

Treatment of melasma should be tailored to each individual, considering the severity of the condition, the patient’s skin phototype, and clinical characteristics. Commonly employed therapies include depigmenting agents like hydroquinone, azelaic acid, kojic acid, and arbutin, which are effective in lightening hyperpigmented patches. Strict photoprotection is crucial to prevent progression and relapses, with the daily use of broad-spectrum sunscreen being recommended. Laser procedures such as Q-switched lasers, Nd: YAG lasers, and intense pulsed light have shown efficacy for refractory cases. Often, combining depigmenting agents, photoprotection, and laser treatments yields the best results [89,90].

Polyphenols have recently gained attention in melasma management. Their mechanisms include neutralizing free radicals to reduce oxidative stress contributing to melanogenesis, modulating inflammatory pathways to alleviate associated inflammation, and potentially inhibiting tyrosinase, a key enzyme in melanin production. While research into polyphenols’ effectiveness in melasma treatment is still developing, initial studies have shown promising results, suggesting that these compounds could significantly manage this complex condition [91,92].

## 3. Clinical Evidence of Polyphenols in Dermatological Diseases

During the last few years, numerous preclinical and clinical studies have highlighted the therapeutic benefits of polyphenols in dermatologic pathologies. These compounds’ mechanistic and pharmacological features indicate their possible influence in acne vulgaris, dermatitis, skin fungal infections, alopecia, and skin cancer pathophysiology [23,24,31,39,70]. Thus, their potential clinical use has become an object of investigation. The following section summarizes key clinical evidence regarding polyphenols’ impact in treating multiple skin disorders (Table 1).

Firstly, green tea is the most extensively investigated polyphenol in acne vulgaris on a clinical level [93,94,100,101,102,103,104,105,106]. In this sense, Kim et al. [93], in a meta-analysis of five randomized controlled studies, reported that the topical application of green tea extract is favorable for acne treatment without significant side effects. Its topical use significantly diminished the cases of inflammatory lesions (−9.38; IC 95%: −14.13 −4.63), the number of inflammatory lesions (−11.39; IC del 95%: −15.91 −6.86), and non-inflammatory lesions (−32.44; IC del 95%: −39.27 −25.62).

Likewise, in a randomized, double-blind, and placebo-controlled trial [106], significant reductions in acne lesions located in the nose, perioral area, and chin were reported after the use of a daily dose of 856 mg of epigallocatechin gallate in women with post-puberty acne. Similarly, Yoon et al. [105] conducted an 8-week randomized, double-blinded, split-face clinical trial, where 1% and 5% epigallocatechin-3-gallate solutions significantly improved inflammatory and non-inflammatory acne lesions. Moreover, Junt et al. [104] reported in a clinical study the significant symptomatic improvement in patients with acne, with reductions in comedones (61%) and blisters (28%) after topical treatment with green tea catechin (polyphenol: 60, 20 mg/mL).

The clinical use of other polyphenols to treat acne vulgaris has also been tested [94,100,101,107]. Waranuch et al. [94], in a single-center, parallel, randomized controlled trial, assessed the anti-acne and anti-blemish properties of a hydrogel formulated with a combination of *Aloe barbadensis* leaf extract, *Garcinia mangostana* peel extract, and *Camellia sinensis* leaf extract. A significant reduction in total acne lesions, severeness index, skin redness, and mean melanin value was reported. Furthermore, in a single-blinded, placebo-controlled, split-face comparative study [107], a considerable reduction in sebum secretions was reported after using green tea lotus extract emulsion.

On the other hand, their potential clinical use in alopecia has gained interest in the last few years. In a randomized controlled placebo study, *Trifolium pratense* flower extract and a biomimetic peptide were applied to thirty patients with mild-to-moderate active HL for four months [96]. A significant increase (46%) in the treated group was found between the anagen/telogen (A/T) relationship. The anagen hair increased by +13%, and telogen hair density decreased by −29% [96]. Similarly, in a randomized controlled placebo study, Loing et al. [96] analyzed the hair-growing activity of procyanidin oligomers in 43 subjects. The treated subjects showed a significantly improved hair density and number of total scalp hair.

Moreover, Seok et al. [108], in a double-masked, controlled, placebo clinical trial, studied the efficacy of *Cistanche tubulosa* and *Laminaria japonica* to promote hair growth. A significant increase in density (test group: 23.29 n/cm^2^ ± 24.26, control: 10.35 n/cm^2^ ± 20.08, *p* < 0.05) and diameter (test group: 0.018 mm ± 0.015, control: 0.003 mm ± 0.013, *p* < 0.05) was found in the hair of the treated group. Lastly, Kamimura et al. [109] in a double-blind clinical study reported the effects of procyanidin B-2 in 29 patients, showing a significant augment in the total number of hair (procyanidin B-2, 6.68 ± 5.53 (mean ± ED)/0.25 cm^2^; placebo, 0.08 ± 4.56 (mean ± ED)/0.25 cm^2^; *p* < 0.005) and terminal hair (procyanidin B-2, 1.99 ± 2.58 (mean ± ED)/0.25 cm^2^; placebo, −0.82 ± 3.40 (mean ± ED)/0.25 cm^2^; *p* < 0.02).

Concerning skin fungal infections, despite a wide range of preclinical studies in the literature about polyphenol’s antifungal activity [41,43,110], the clinical level of the data is very limited [95,111,112]. In this sense, Ikeda et al. [95], in a double-masked, randomized, placebo-controlled trial, observed a significant improvement in tinea pedis. Similar results were reported in diabetic patients with onychomycosis [113]. *Solanum chrysotrichu* has proven to be another useful phyto drug [112,114]. Herrera-Arellano et al. [114], in a controlled and randomized double-masked clinical trial, demonstrated a clinical efficacy of 92.16% in *pityriasis capitis* patients after the use of the extract. In addition, it has been reported that Brazilian green propolis causes a clinical improvement in tinea pedis interdigitalis, tinea corporis [115], tinea capitis, and tinea versicolor [116]. Finally, other polyphenols such as *Ocimum gratissimum* [117], *Acalypha wilkesiana* [118], *Cymbopogon citratus* [119], *Cassia alata* [120], and *Melaleuca alternifolia* [121] have demonstrated improvement in fungal infections.

On the other hand, various clinical studies assessed the efficacy of polyphenols in different types of dermatitis, obtaining mixed results. Two clinical trials related to atopic dermatitis (AD) were found: the first is a monocentric, randomized, controlled, double-blind study [122] that evaluated the efficacy and safety of a vitamin E, gallate, epigallocatechin, and grape seed procyanidin (MD2011001) topical cream in a sample of patients with mild-to-moderate AD for 28 days, in which a statistically significant difference was not observed when compared to placebo.

In a second randomized, double-masked, controlled, placebo trial performed by Mehrbani et al. [98], 52 adult subjects with mild-to-moderate AD were treated with lyophilized powdered milk serum rich in flavonoids, quercetin, kaempferol, and rutin or placebo [98]. The results were promising, with statistically significant improvement compared to placebo, regarding clinical manifestations such as elasticity, hydration, pruritus of affected skin, and diminished sleep disturbances; moreover, it was concluded that these improvements were prolonged 15 days after the treatment. Nevertheless, the data provided by these investigations do not allow us to individually assess the effect of polyphenols on AD [123].

Radiation dermatitis (RD) in oncologic patients has been a focus of polyphenol research. Several studies assessed topical epigallocatechin-3-gallate (EGCG), an active compound of green tea, to prevent and treat this affection. In the research carried out by Zhao et al. [99], the effect of EGCG in RD prevention was evaluated via a second phase, randomized, double-blind, controlled, placebo trial and performed in breast cancer patients treated with postoperative radiotherapy. Following EGCG treatment, the incidence, severity, and clinical manifestations showed a statistically significant decrease. Moreover, the same authors previously tested the tolerability of the therapy in a phase I clinical trial [124], where no significant increase in adverse effects was observed. In addition, a more recent phase I clinical trial by Xie et al. [125] evaluated the use of EGCG for RD in patients with thoracic cancer, observing improvement in sensibility, pruritus, redness, pain, burning, and traction sensations, with no adverse effects reported.

EGCG is not the only polyphenol studied for RD; silymarin, a flavonoid extracted from *Silybum marianum*, was assessed by Karbasforooshan et al. [126] in a randomized, double blind and controlled placebo clinical trial. In the study, 1% silymarin gel was applied to breast cancer patients for RD prevention. Even though all subjects developed RD by the end of the study, a delayed onset and decreased severity were observed in the silymarin group.

Furthermore, polyphenols are potential therapeutic agents for contact dermatitis (CD). Improvements in inflammation markers were observed, specifically lower levels of INFγ, IL-4, IL-17, pentraxin 3, and NO and augmented levels of IL-10, in women with CD treated with 300 mg of polyphenols per day extracted from red grape seeds [127].

Lastly, regarding the use of polyphenols for treating and preventing skin cancer, even though a growing body of in vitro and in vivo experimental studies with promising results exist, currently, no clinical trials assessing the safety and efficacy of these compounds is available [128].

## 4. Bioavailability of Polyphenols and Routes of Administration

The bioavailability of polyphenols is a crucial issue in their therapeutic application. Despite their high bioactivity, the performance of polyphenols in vivo may be limited due to low water solubility, low lipophilicity, and inadequate molecular size; therefore, polyphenols suffer from structural instability in biological environments. All these characteristics lead to poor biodistribution, first-pass metabolism, poor penetration, and accumulation in the different organs [129,130].

Nowadays, topical administration has become one of the most attractive routes for the pharmaceutical industry. This method of administration can overcome some of the drawbacks of oral administration, such as low bioavailability, while ensuring the continuous and stable release of the polyphenol at the site of action. However, normal skin possesses a formidable barrier to drug absorption, mainly due to its particular lipid composition and the stratum corneum tissue [129].

Nanomedicine represents a favorable tool to increase the bioavailability and activities of natural products [131]. Nanostructured delivery systems that allow for a more effective delivery of polyphenols to the skin are currently being developed [130]. Nanocarriers can improve the stability, bioavailability, and targeted delivery of active ingredients, allowing better penetration and efficacy in treating specific skin conditions [132,133,134,135,136].

## 5. Natural Components Other than Polyphenols Being Used in Skin Conditions

While polyphenols represent a promising alternative in various dermatological therapies, other organic compounds, including terpenes and terpenoids, carotenoids, and benzoquinones, also exhibit beneficial properties for skin conditions [137,138,139].

Terpenes and terpenoids are a broad class of organic compounds found in several plants, as well as in some insects and microorganisms. Due to their anti-inflammatory, anti-microbial, and anti-tumor effects, terpenoids have been studied for their possible effects on inflammatory skin conditions, *Staphylococcus Aureus* infections, impetigo, dermatomycosis, and in the prevention and treatment of skin cancer [137,140]. Fungal terpenes and terpenoids have demonstrated tyrosinase inhibition, suggesting their potential use in treating skin pigmentation disorders and melanogenesis-related cancers [141,142,143,144].

On the other hand, carotenoids constitute a specific type of terpenoid; within this classification, there are compounds such as lutein, canthaxanthin, α-carotene, β-carotene, and lycopene [145]. These molecules possess anti-inflammatory, anti-tumor, and antioxidant properties [138]. Recent interventional studies have proven the effects of lutein on UV light-induced carcinogenesis in animal models, as well as its beneficial effects on physiological skin parameters in humans, including antioxidant protection against UV light irradiation [146,147,148]. Similarly, clinical trials have confirmed the antioxidant activity of carotenoids and their capacity to improve erythematous affections. [149,150]. Moreover, canthaxanthin has demonstrated its utility in erythropoietic protoporphyria, a genetic disorder characterized by sun sensitivity [151].

Benzoquinones represent a different class of organic compounds, widely distributed in fungus, bacteria, arthropods, and plants. Embelin is a benzoquinone produced by the plant *Embelia ribes*, known by its pharmacological and therapeutic effects, including antioxidant, anti-inflammatory, and anti-tumor properties [152]. Numerous research studies have managed to show its capacity to inhibit leukocyte aggregation, suppressing TNF and IL-1, along with other inflammatory factors, and this ability might be useful in skin conditions such as psoriasis and dermatitis. Simultaneously, a variety of studies have pointed out the pro-apoptotic, anti-proliferative, anti-metastatic, anti-angiogenic, and anti-inflammatory qualities of the said compound, thus, representing a promising alternative for skin cancer treatment and prophylaxis [152,153,154].

Many natural sources with potential skin benefits remain underexplored. Future studies should prioritize the research of lesser-known plant extracts, marine compounds, and other natural resources to identify novel bioactive ingredients and their therapeutic role in skin disorders.

## 6. Conclusions

Dermatologic disorders continue to be among the most frequently encountered and challenging medical conditions in contemporary medicine. Due to their intricate and multifaceted pathophysiological mechanisms, researchers have been actively pursuing and evaluating novel therapeutic alternatives for their management over the past several decades. Within this evolving therapeutic landscape, polyphenols have emerged as a particularly promising group of metabolites, distinguished by their remarkable ability to produce a diverse array of beneficial effects—including potent antioxidant, anti-inflammatory, antimicrobial, antitumoral, and DNA-repairing properties—through multiple cellular and molecular mechanisms in various dermatological conditions.

In this dynamic context, the extensive body of preclinical evidence supports and validates the potential therapeutic applications of polyphenols in addressing the underlying pathogenic processes of numerous dermatological conditions, including but not limited to acne vulgaris, various forms of dermatitis, cutaneous fungal infections, different patterns of alopecia, and several types of skin cancer. Despite these positive preclinical findings, the current landscape of clinical trial evidence remains notably limited in scope and depth. Therefore, there exists a pressing need for additional high-quality clinical research studies characterized by larger patient populations, extended follow-up observation periods, and more rigorous methodological frameworks to comprehensively evaluate and definitively establish the clinical efficacy and practical applications of polyphenol-based interventions in the treatment of dermatological disorders. Furthermore, the development of novel approaches dedicated to boost the natural properties of these molecules by enhancing characteristics such as absorption, solubility, and bioavailability, among others, represents a promising field of research in modern phytotherapy.

## Figures and Tables

**Table 1:** Summary of key clinical evidence regarding polyphenols and skin disorders.

Author	Skin Disorder	Methodology	Results
Kim et al. [93]	Acne vulgaris	Meta-analysis with five randomized controlled clinical trials evaluated the efficacy and safety of green tea extract for treating acne.	Green tea extract significantly reduced the number of inflammatory lesions (−9.38; 95% CI: −14.13 to −4.63), inflammatory lesion counts (−11.39; 95% CI: −15.91 to −6.86), and non-inflammatory acne lesions (−32.44; 95% CI: −39.27 to −25.62).
Waranuch et al. [94]	Acne vulgaris	A single-center, parallel, randomized controlled trial assessed the anti-acne and anti-blotch activity of a hydrogel formulated with a combination of *Aloe barbadensis* leaf extract and *Garcinia mangostana* peel extract.	There was a reduction in total acne lesions (*p* < 0.0001) and mean acne severity index. Also, a decrease in skin redness (*p* < 0.05) and in mean melanin value (*p* = 0.037) was found.
Ikeda et al. [95]	Skin fungal infection	A double-blind, randomized, placebo-controlled trial evaluating the effects of a foot bath containing green tea polyphenols in patients with interdigital tinea pedis.	The use of a foot bath containing green tea polyphenols for 12 weeks produced a significant reduction in the size of the affected area (*p* < 0.001).
Loing et al. [96]	Alopecia	A randomized, placebo-controlled study assessed the efficacy of *Trifolium pratense* flower extract and a biomimetic peptide in alopecia.	The anagen/telogen (A/T) ratio increased by +46%, anagen hair increased at an average of +13%, and telogen hair density decreased by −29% after 4 months of treatment.
Takahashi et al. [97]	Alopecia	A double-blind, randomized, placebo-controlled trial evaluated the efficacy of the external application of 0.7% apple procyanidin oligomers in patients with pattern baldness.	There was a significant increase in total number of hair (procyanidin, 3.3 ± 13.0 (mean ± SD)/0.50 cm^2^; placebo, −3.6 ± 8.1/0.50 cm^2^; *p* < 0.001, two-sample *t*-test).
Mehrbani et al. [98]	Atopic Dermatitis	A double-blind, randomized, placebo, controlled trial assessed the effect of powdered lyophilized milk serum with cuscuta extract to treat AD.	A marked increase in skin humidity and elasticity was observed (*p* < 0.001), along with diminished pruritus (*p* < 0.05) and sleep disturbances (*p* < 0.05).
Zhao et al. [99]	Radiation Dermatitis	A phase II, double-blind, randomized, controlled clinical trial was performed to determine the therapeutic and preventive properties of EGCG on RD patients with breast cancer treated with post-operatory radiotherapy.	EGCG significantly reduced the incidence, severity, and clinical manifestations of RD.

Abbreviations: A/T: anagen/telogen; AD: atopic dermatitis; RD: radiation dermatitis; EGCG: epigallocatechin-3-gallate topical serum.

## Abbreviations

The following abbreviations are used in this manuscript:

GBD	Global Burden Disease
SD	Skin disorders
AV	Acne vulgaris
ROS	Reactive oxygen species
TPCLE	Total phenolic content leaf extract
IL-8	Interleukin-8
IL-1β	Interleukin-1β
TNFα	Tumor Necrosis Factor Alpha
MAPK	Mitogen-Activated Protein Kinase
NF-kB	Nuclear Factor Kappa-Light-Chain-Enhancer of activated B cells
MPP-9	Matrix Metalloproteinase 9
TLR2	Toll-like receptor 2
mRNA	Messenger RNA
NOS	Nitrous Oxide
ICAM-1	Intercellular Adhesion Molecule-1
COX-2	Cyclooxygenase-2
COX-1	Cyclooxygenase-1
JNK	c-Jun N-terminal kinase
KAS-III	3-Ketoacyl ACP synthase
QML	Quercus mongolica leaf extract
PD	Pedunculagin
IL-6	Interleukin-6
PP	Pomegranate peels
5αAR-1	5α reductase type 1
BK	Bokusoku
AD	Atopic dermatitis
TCS	Topical corticosteroids
CI	Calcineurin inhibitors
IL-4	Interleukin-4
ERK	Extracellular Signal-Regulated Kinase
IgE	Immunoglobulin E
TSLP	Thymic Stromal Lymphopoietin
TTLE	*Tambourissa trichophylla* leaf extract
AP	Acacia polyphenol
STB	*Schizonepeta tenuifolia* Briquet
AOM	*Alpinia oxyphylla* Miquel
CD	Contact dermatitis
mTOR	Mammalian Target of Rapamycin
TLR4	Toll-like receptor 4
SIRT1	Sirtuin 1
IGF-1	Insulin-like Growth Factor 1
KGF	Keratinocyte growth factor
TERT	Telomerase reverse transcriptase
VEGF	Vascular Endothelial Growth Factor
DPC	Dermal papilla cell
PDGF	Platelet-derived Growth Factor
EGF	Epidermal Growth Factor
DHT	Dihydrotestosterone
GA	Gallic acid
EA	Ellagic acid
CYP51	Sterol 14α-demethylase P450
SE	Squalane Epoxidase
CA	Caffeic Acid
LicoA	Licochalcone A
FAS	Fatty acid synthase
CDK	Cyclin-Dependent Kinase
ATP	Adenosine triphosphate
MMP	Mitochondrial membrane potential
ISO	Isoquercitrin
SOD	Superoxide Dismutase
5-LOX	5-Lipooxygenase
HL	Hair loss
AGA	Androgenic alopecia
HF	Hair follicle
TE	Telogen effluvium
H_2_O_2_	Hydrogen peroxide
ALP	Alkaline phosphatase
ORSC	Outer root sheath cells
PM	Polygonum multiflorum
TSG	2,3,5,4-Tetrahydroxystilbene-2-*O*-β-d-glucoside
AR	Androgenic receptor
UV	Ultraviolet
NMSC	Non-Melanoma Skin Cancer
BCC	Basal Cell Carcinoma
SCC	Squamous cell carcinoma
MSC	Melanoma Skin Cancer
CPD	Cyclobutane pyrimidine dimer
NER	Nucleotide Excision Repair
PI3K	Phosphatidylinositol 3 Kinase
AKT	Kinase protein B
TP	Tea polyphenol
ETR	Erythematotelangiectatic Rosacea
PPR	Papulopustular Rosacea
EGCG	Epigallocatechin gallate
RD	Radiation dermatitis
INFγ	Interferon-Gamma
IL-17	Interleukin-17
IL-10	Interleukin-10
